# Re-Evaluation of the AASHTO-Flexible Pavement Design Equation with Neural Network Modeling

**DOI:** 10.1371/journal.pone.0113226

**Published:** 2014-11-14

**Authors:** Mesut Tiğdemir

**Affiliations:** Department of Civil Engineering, Süleyman Demirel University, Engineering Faculty, Isparta, Turkey; Beihang University, China

## Abstract

Here we establish that equivalent single-axle loads values can be estimated using artificial neural networks without the complex design equality of American Association of State Highway and Transportation Officials (AASHTO). More importantly, we find that the neural network model gives the coefficients to be able to obtain the actual load values using the AASHTO design values. Thus, those design traffic values that might result in deterioration can be better calculated using the neural networks model than with the AASHTO design equation. The artificial neural network method is used for this purpose. The existing AASHTO flexible pavement design equation does not currently predict the pavement performance of the strategic highway research program (Long Term Pavement Performance studies) test sections very accurately, and typically over-estimates the number of equivalent single axle loads needed to cause a measured loss of the present serviceability index. Here we aimed to demonstrate that the proposed neural network model can more accurately represent the loads values data, compared against the performance of the AASHTO formula. It is concluded that the neural network may be an appropriate tool for the development of databased-nonparametric models of pavement performance.

## Introduction

The AASHO road test was probably the most significant piece of pavement research performed in the 20^th^ century. The results of the AASHO road test have served as the basis for nearly all subsequent pavement designs used in the original construction of the interstate highway system (IHS) after 1961. When considering the overall performance of HIS pavements, we find that most of the pavements have lasted the expected 20 years while carrying traffic volumes far in excess of those predicted at the time of design [1].

The AASHO road test, possibly the largest and most successful controlled civil engineering experiment ever undertaken, was conducted about 50 years ago. The results of the study are still widely used across the world. Significant results from this road test still govern pavement design worldwide, including in areas such as: (a) equivalent single-axle loads (ESALs); (b) the serviceability–performance concept; (c) effects of layer thickness and strength; and (d) effectiveness of dowels and joint spacing. In addition, AASHO road test also changed the way that pavement research is conduct by illustrating the power of factorial experiments, high-quality data, and statistical analysis [2].

The American Association of State Highway Officials (AASHO) road test at Ottawa, Illinois provided the basis for calculating the required pavement thickness. Models were developed that related pavement performance, vehicle loadings, the strength of roadbed soils, and the pavement structure. Equation 1 is the AASHTO equation used for design purposes. The purpose of the AASHTO model in the pavement thickness design process is to calculate the required structural number (SN). This is the strength of the pavement that must be constructed to carry the mixed vehicle loads over the roadbed soil, while providing satisfactory serviceability during the design period. Knowing the SN, the pavement layer thickness or overlay thickness can be calculated [3].

Vehicle loads are expressed in 18-kip equivalent single axle loads (18-kip ESAL). This information is normally generated by the district planning office.
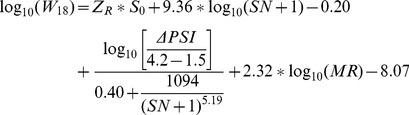
(1)The unknown to be determined is:

SN = Structural number required inches.

The inputs include the variables:

W_18_ = Accumulated 18-kip ESAL over the life of the project.
Z_R_ = Standard normal deviate.
M_R_ = Resilient modulus psi

The inputs include the constants:

S_O_ = Standard deviation.
ΔPSI = Change in serviceability.

In the strategic highway research program (SHRP) project [4], as the equation was being used for research rather than design, a 50% reliability was selected as appropriate for mean predictions. At 50% reliability ZR = 0, and this term drops out of the equation. Equation 1 was used to predict the total KESALs (1000 ESALs) required to cause the observed losses in PSI.

Resilient moduli for the subgrade (Mr) were estimated based on the procedure provided in the 1986 guide. It should be noted that this procedure does not consider seasonal effects, so the subgrade moduli were not entirely consistent. However, the differences in magnitudes that would have occurred from seasonal adjustments would not have made significant differences to the results. Historical traffic data provided by the State Highway Agencies (SHAs) were used for the traffic data (W) in these calculations. The cumulative KESALs for each section were divided by the number of years since the test section was opened to traffic to obtain average values per year. This allowed extrapolation of an addition one or two year beyond 1989, to estimate a traffic level associated with the date of performance monitoring activities. Most of the monitoring data used were obtained in 1990 or 1991.

Banan and Hjelmstad [5] re-examined the AASHO road-test data, using adaptive random partitioning neural-network model developed by Banan and Hjelmstad (1995), and show that neural network model can represent the data far better than the AASHO formula. They conclude that the neural network may be an appropriate tool for the development of data-based models of pavement performance in the future.

In the report of FHWA-HRT-06-109, Souza et al. [6] presents an analysis between the international roughness index (IRI) and the standard deviation of longitudinal roughness (s), as well as a neural network study developed to predict the critical level of roughness. Using suitable software, the IRI and s values were computed for every longitudinal pavement profile measured. The neural network could forecast the IRI with an extremely high correlation factor (R^2^ = 0.99).

Ozgan used the artificial neural network method to model the Marshall Stability (MS) of asphalt concrete under varying temperature and exposure times [7]. Alavi et al., derived a high-precision model to predict the flow number of dense asphalt mixtures using a generalized regression neural network and multiple regression-based analyses [8]. Kok and et al., aimed to model the complex modulus of base and styrene–butadiene–styrene (SBS) modified bitumens by using artificial neural networks (ANNs). They used the variants of the Levenberg–Marquardt, scaled conjugate gradient and Pola-Ribiere conjugate gradient algorithms [9]. Wu and et al., studied the computation of the stress intensity factors at the crack tip for pavement crack propagation analysis using a neural network approach based on semi-analytical finite element analysis [10]. Kok and et al., studied to model the complex modulus of styrene–butadiene–styrene modified bitumen samples by different methods using artificial neural networks [11].

The principal goal of this study was to relate the design life of the pavements obtained from the AASHTO equation, which is known worldwide, with the observed life in terms of equivalent single-axle loads.

## Methods

A neural network is a massively parallel distributed processor that has a natural propensity for storing experiential knowledge and making it available for use. It resembles the brain in two respects [12]:

1. Knowledge is acquired by the network through a learning process.
2. Interconnection strengths known as synaptic weights are used to store the knowledge.

At its most basic, learning is a process by which the free parameters (i.e., synaptic weights and bias levels) of a neural network are adapted through a continuing process of stimulation by the environment in which the network is embedded. The type of learning is determined by the manner in which the parameter changes take place. In a general sense, the learning process may be classified as [13]: Learning with a teacher (also referred to as supervised learning); or learning without a teacher (also referred to as unsupervised learning).

**Figure 1:**
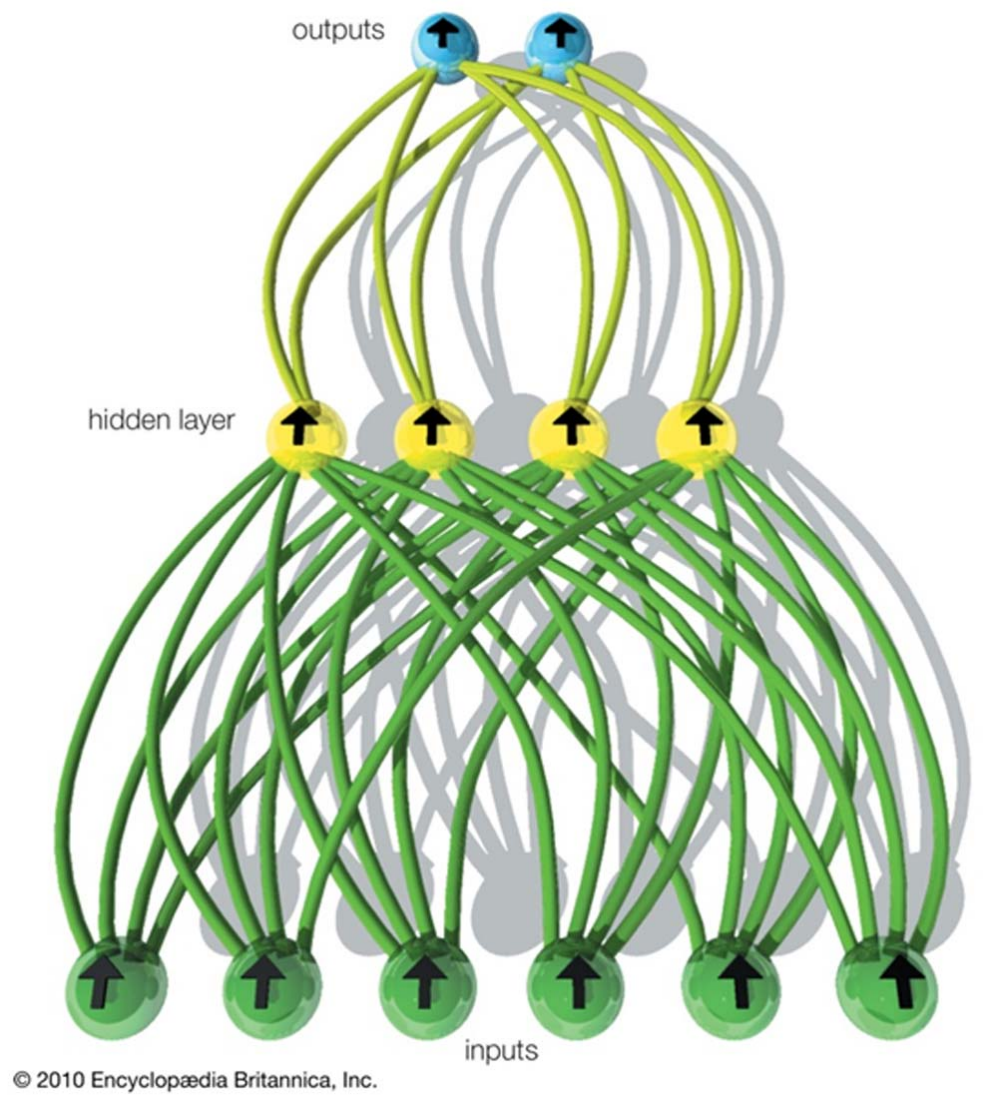
A simple feed-forward neural network [15].

Learning is thus a development from the simple Delta rule in which extra hidden layers (layers additional to the input and output layers, not connected externally) are added. The network topology is constrained to be feed-forward (i.e., loop-free); generally connections are allowed from the input layer to the first (and possibly only) hidden layer; from the first hidden layer to the second, and so no until progressing from the last hidden layer to the output layer (Figure 1) [14]–[15].

The hidden layer learns to recode (or to provide a representation for) the inputs. More than one hidden layer can be used. This architecture is more powerful than single-layer networks as it can be shown that any mapping can be learned, given two hidden layers (of units). The units are generally more complex than those in the original perceptron and their input/output graph can be summarized as the function:
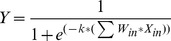
(2)


The weight change rule is a development of the perceptron learning rule. Weights are changed by an amount proportional to the error at that unit multiplied by the output of the unit feeding into the weight.

Running such a network consists of several components, including: Forward pass, where the outputs are calculated and the error at the output units calculated; backward pass, where the output unit error is used to alter weights on the output units. Subsequently, the errors at the hidden nodes are calculated (by back-propagating the error at the output units through the weights), and the weights on the hidden nodes are altered accordingly.

For each data pair to be learned a forward pass and backwards pass is performed. This is repeated until the error is acceptably low.

### Comparisons of Predicted Versus Observed Traffic

With data from 244 general pavement studies (GPS)-I and GPS-2 of in-service flexible pavement test sections across the USA, the Long Term Pavement Performance (LTPP) database offers an unprecedented opportunity for evaluating the ways in which flexible pavements are designed and their associated performance. In these analyses of the SHRP LTPP database, all efforts were concentrated on evaluating the AASHTO pavement design equation and the suitability of the data collected from these test sections for use in such evaluations. From these evaluations, it has been established that the existing AASHTO flexible pavement design equation does not accurately predict the pavement performance of the SHRP LTPP test sections, and unfortunately, generally predicts many more ESALs needed to cause a measured loss of present serviceability index (PSI) than the pavements had actually experienced. Many explanations have been identified. Although modifications have been made over the years to expand the inference space of these design equations, any such modifications cannot be without their own limitations [4].

From SHRP long-term pavement performance (LTPP) studies, it is evident that environmental properties such as rainfall, freezing index, and freeze-thaw cycles have a greater impact on pavement performance than that accommodated by the AASHTO flexible pavement design equation.

**Figure 2:**
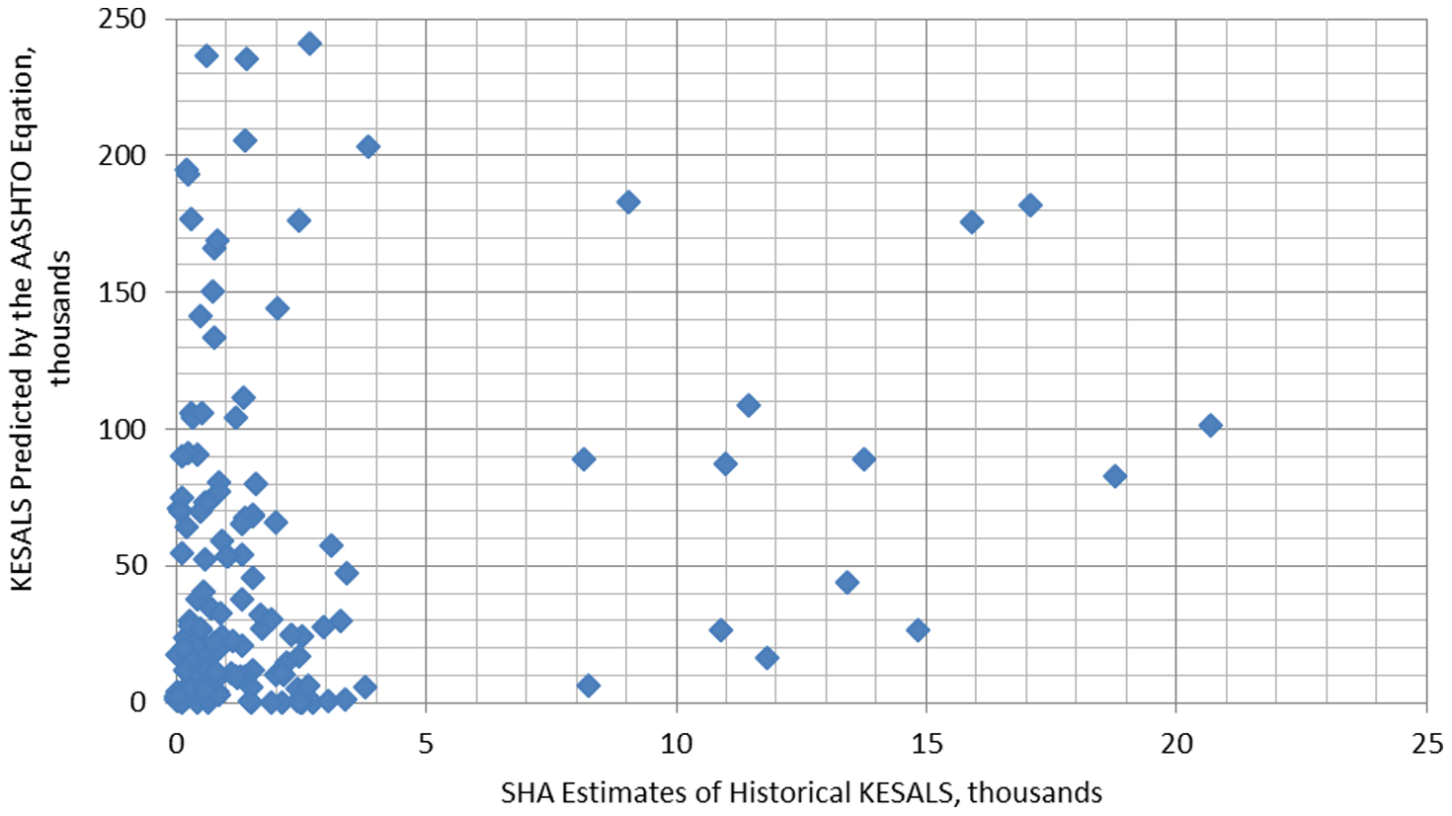
SHA Estimates of Historical Traffic Versus AASHTO Predicted Traffic.

**Figure 3:**
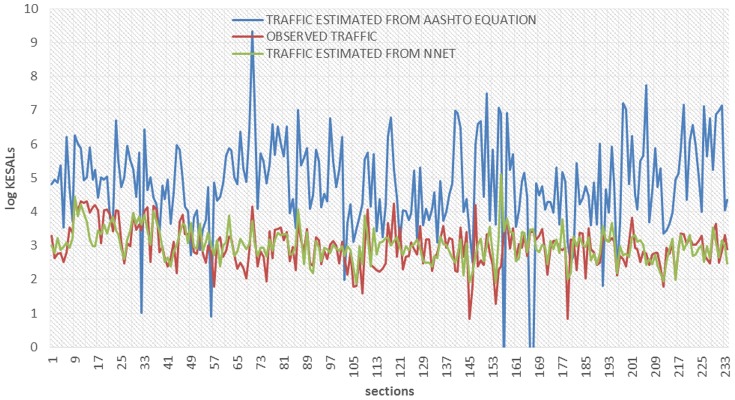
Predicted KESALs values by AASHTO equation, NNET model, and the relations of them to the observed values.

Figure 2 provides a plot of predicted KESALs versus those estimated by the SHA's throughout 1989 and extrapolated through 1991. As can be seen, the traffic predicted by the AASHTO equation is consistently much higher than the estimates of historical traffic provided by the SHAs. Only nine of the 244 predictions were lower than the estimates of the state highway agencies. Almost half of the estimates (in 112 sections) predicted traffic levels 100-fold greater than the SHA estimates (Figure 3). Note that the average ratio (8770) and standard deviation (51,800) are distorted by several sections where this ratio exceeded 100,000. This extreme lack of fit of the design equation to the in-service data is not entirely due to the shortcomings of the equation itself. Limitation of the input data are also believed to have contributed to the apparent differences between predicted and estimated ESALs. The future availability of ESALs estimates that would include some years of measured data, plus higher [resent serviceability index values should allow a somewhat more accurate evaluation of the deficiencies in the equation itself.

### Prediction of the observed traffic (KESALs) using neural networks

In this study, different network architectures have been employed to model the database. The regression coefficients were given in Table 1 for the output of KESALs estimated by the AASHTO pavement equation. As a result of the comparison of the network architectures, the feed forward-back propagation network architecture was chosen for this study.

**Table 1:** Regression coefficients for the different network architectures.

NETWORK	LOOP	NEURON	R	NEURON	R	NEURON	R
FEED FORWARD BACKPROP.	1000	5	0.744	10	**0.923**	20	**0.968**
CASCADE FORWARD BACKPROP.			0.785		0.886		**0.972**
ELMAN BACKPROP.			0.707		0.699		0.688

training:Levenberg-Marquardt, performance: mean squared error.

The previously described AASHTO road test database was initially analyzed using the feed-forward/back-propagation neural network modeling system with the objective of finding an NN model as a function of the available input parameters. A single output layer for the KESALs value was used. The input layer consisted of 7 data neurons as follows:

Input 1: rain = average annual rain fall
Input 2: avg32 = average annual number of days below 32 F (0 C)
Input 3: drainage coefficient (m) = (1.2−0.006*average rain) * (1.2−0.006*%−200)
Input 4: observed PSI loss = (initial PSI)−(calculated PSI)
Input 5: back-calculated mr = [(fwd load)*0.2792)/(deflection at 60″) * 60]
Input 6:SN = .44*AC+.34*BBB+.23*NBBB+m*(.14*UBB+.07*SUBB+.15*SS)

Asphalt (AC), Bit Bound Base (BBB), Non Bit Bound Base(NBBB), Unbound Base(UBB), Sub-base (SUBB), Stab-Subgrade (SS)

Input 7: The subgrade type = Gravel, Sand, Silt, Clay, Silty Clay, Rock

The subgrade type was divided into 6 types according to the unified soil classification system (SUCS), plus rock foundation. The following codes were attributed to input of the subgrade type: Gravel = 1; Sand = 2; Silt = 3; Clay = 4; Silty Clay = 5; and Rock = 6.

Two neural network models have been employed. In the first model, KESALs predicted by the AASHTO equation as log10(KESALs)AASHTO was used as the output. In order to obtain the AASHTO KESALs traffic value without using the design equation, the first neural network model was developed. Owing to the large differentiation between the observed and AASHTO KESALs, in the second model, the output was chosen to be the observed KESALs values. However, the model was not able to present the database as expected. Hence, the output was accepted as the ratio of logarithmic KESALs by AASHTO to logarithmic observed KESALs value.

**Figure 4:**
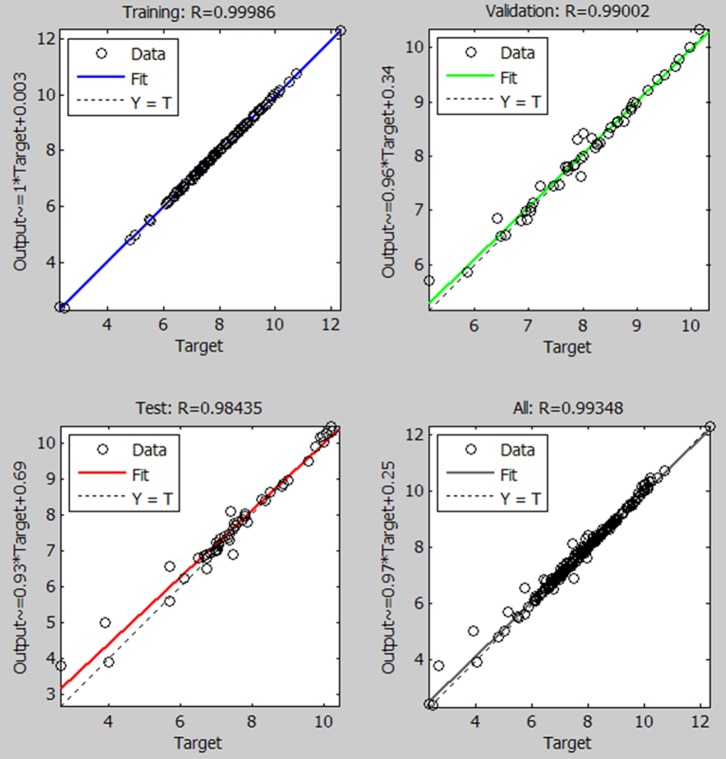
Regression plot of KESALs predictions for target (AASHTO Equation produced) with model output of ANN (7 inputs).

Because of an absence of rain data for some sections, 234 of a total of 244 sections were employed for modeling. The training data-set, comprising 164 random sections of 234 available (70%), was initially chosen for the learning stage. The NN model produced excellent results, as illustrated in Figure 4, which shows the training targets (predicted KESALs by AASHTO equation) and the network outputs (computed KESALs values). The correlation coefficient for the training stage was high (R^2^ = 0.999). Of the remaining 30% of the data-set, 15% of the sections were used to validate the model and another 15% were used to test the model. Predictions were good despite a higher dispersion than in the learning stage. The correlation coefficient for the validation stage was R^2^ = 0.990.

**Figure 5:**
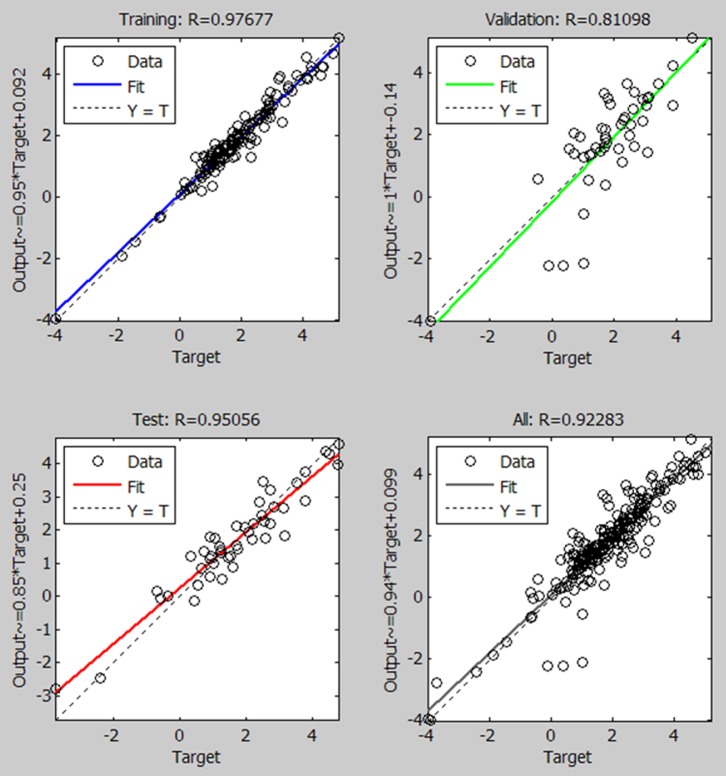
Regression plot of KESALs ratio (log10[AASHTO/actual KESALs]) predictions for target with model output of ANN (7 inputs).

**Figure 6:**
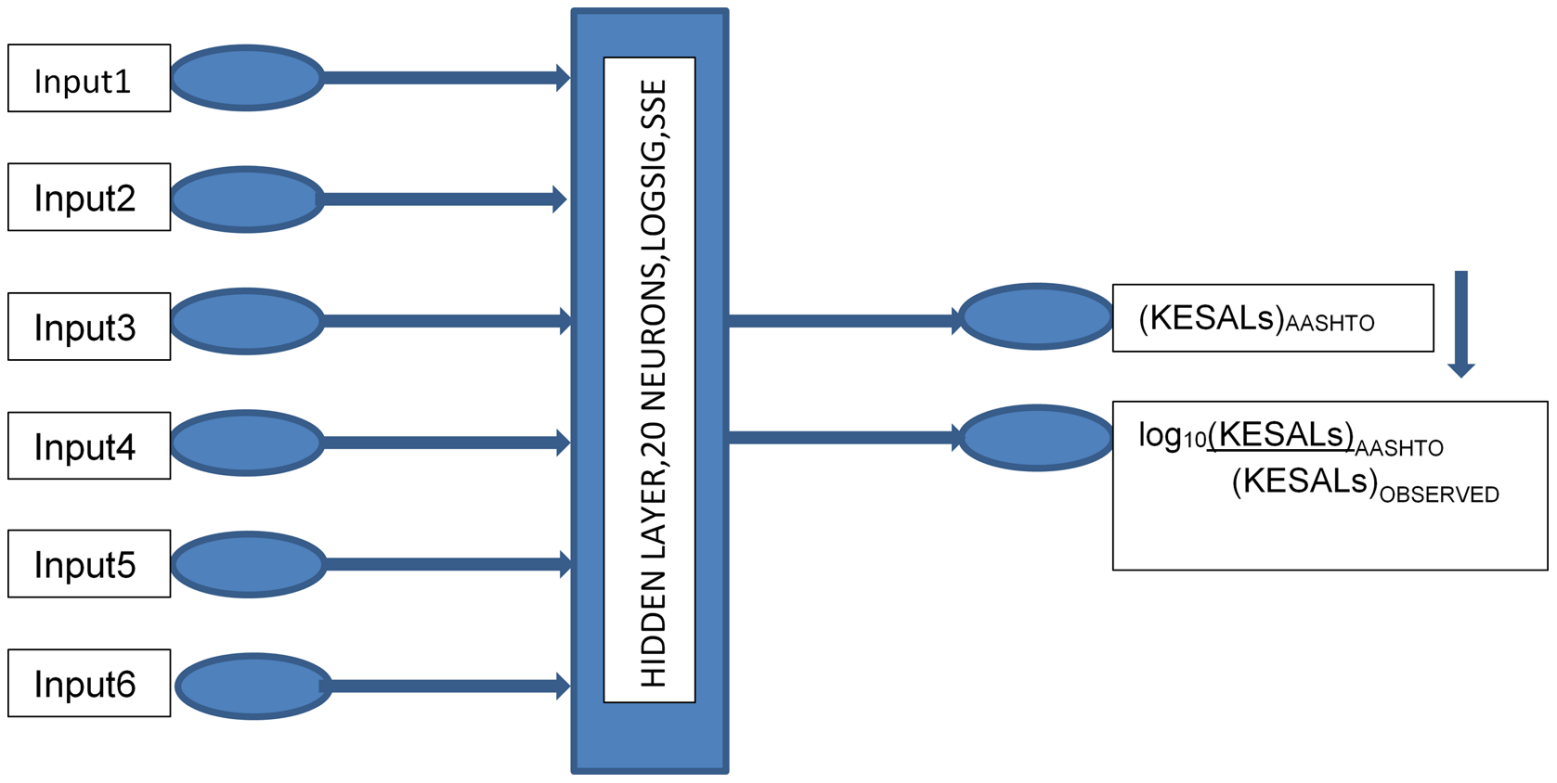
The proposed NN model combined of the NN1 and NN2 models.

**Figure 7:**
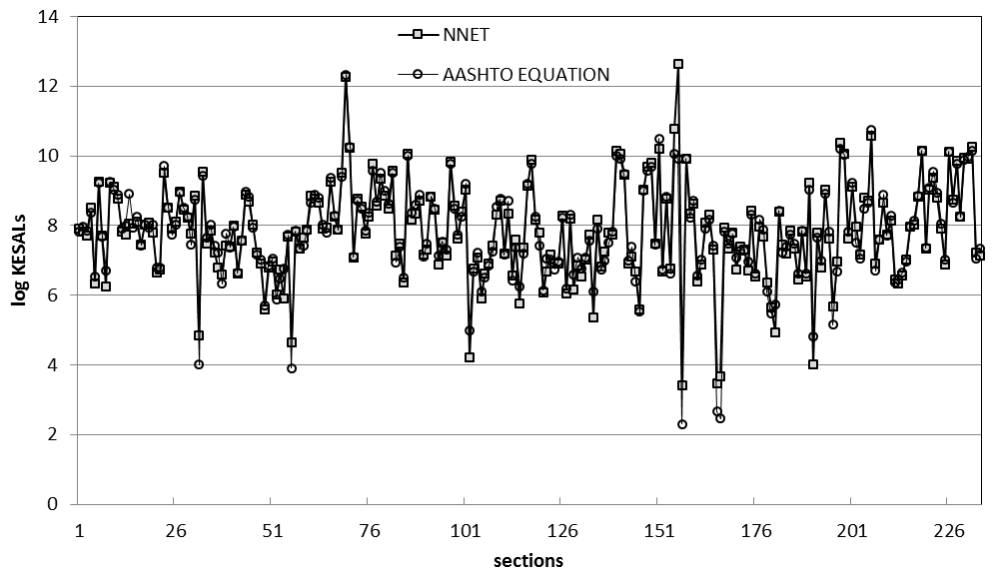
AASHTO equation modeling with neural networks (correlation of 0.97).

**Figure 8:**
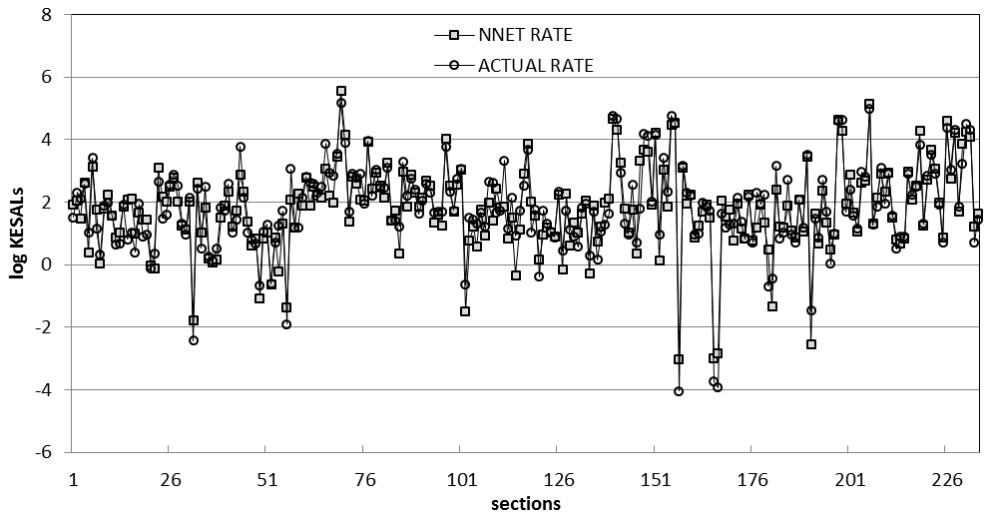
The ratio of KESALsAASHTO to KESALsOBSERVED modeling with neural networks (correlation of 0.94).

In the second model, the output was chosen to be the logarithmic ratio of KESALs calculated by the AASHTO design equation to observed values. The inputs were the same as the first model of 7 inputs. The training, the validation, and the test databases were the same as used above. The results are shown in Figure 5. With this model, for inputs of road test data, the output will be given as the ratio of calculated KESALs to those observed, and the observed value may therefore be predicted as follows:

The output of NN model:
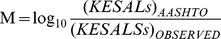
(3)This neural network model can easily give the KESALs value without using the AASHTO complex pavement design equation. At the same time, the model gives the ratio M, hence the observed values can be predicted as:
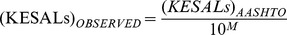
(4)Here, based on the previous two NN models, a new combined model that contains two outputs is proposed. The inputs are the same as in previous studies. The first output is the KESALs predicted by the AASHTO design equation. This value is estimated by the combined NN model using the inputs of road test data. In parallel, the second output of the model is estimated as the logarithmic ratio of the KESALs by the AASHTO equation to observed KESALs. Thus, the observed KESALs may be estimated using Equation 4. Figure 6 shows the proposed combined NN model. Scatter plots between targets and estimations by the NN model are given for both output 1 (i.e., KESALs obtained using the AASHTO design equation) and output 2 (i.e., the logarithmic ratio between predicted and observed KESALs) with correlations of 0.97 and 0.94, respectively (Figure 7, 8).

The developed NN model first predicts the output value of the AASHTO design equation and, second, gives the ratio of this estimated value to the actual observed value. Using this ratio, the actual KESALs value may be estimated.

**Figure 9:**
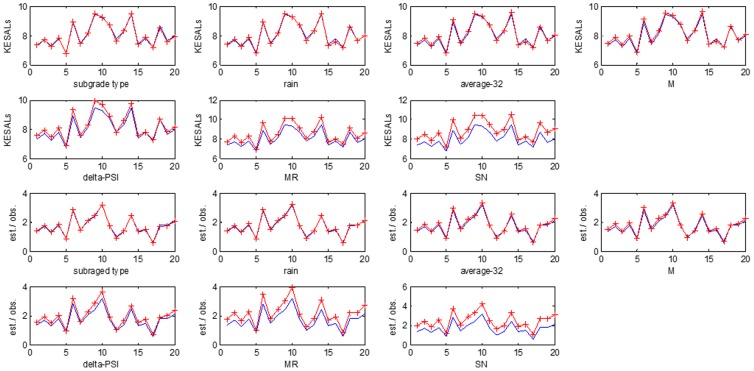
The sensitivity analysis of relative contributions of each input parameters to the outputs.

A sensitivity analysis was performed to determine the relative contribution of each input parameters to the estimation of the output. The outputs are KESALs and the ratio of estimated KESALs to observed KESALs. This analysis have been performed for the change of +10% of all inputs' individually for the twenty sections. The inputs of the delta-PSI, the MR, and the SN give the most relative contribution to the estimation of outputs (Figure 9).

### Prediction of KESALs Using Multivariate Statistical Models

The seven inputs of the same road test database were used to attempt to assess the performance of the established statistical models for predicting the actual KESALs, as well as those predicted using the AASHTO equation. The multivariate linear models were tested. The results of the database statistical analyses are given in Table 2–3. No correlation could be found (R^2^ = 0.23) of the linear model for observed KESALs values. Whereas the correlation is about R^2^ = 0.68 for predicted KESALs using the AASHTO equation. The stepwise analysis was employed to model the database. The results show highly significant (P<0.0001) inputs on the outputs as the delta-PSI, MR, and the SN values according to the statistical significance (P, 2 tail) analysis, as seen before in the sensitivity analysis.

**Table 2:** Statistical analysis of the database.

Effect	Coefficient	Std Error	Std Coef	Tolerance	t	P(2 Tail)
CONSTANT	3,944	0,191	0,000	,	20,697	0,000
AVG32	0,002	0,001	0,072	0,835	1,745	0,082
DELTAPSI	1,335	0,111	0,479	0,886	11,981	0,000
MR	0,000	0,000	0,366	0,995	9,696	0,000
SN	0,428	0,032	0,520	0,939	13,392	0,000

Dep Var: KESALS N: 234 Multiple R: 0,822 squared multiple R: 0,676.

Adjusted squared multiple R: 0,670 Standard error of estimate: 0,791.

**Table 3:** Analysis of Variance.

Source	Sum-of-Squares	df	Mean-Square	F-ratio	P
Regression	298,208	4	74,552	119,195	0,000
Residual	143,232	229	0,625		

## Conclusions

In this paper, databased mathematical-neural network models of long-term pavement performance studies have been obtained by re-evaluating the AASHO road-test data.

Two neural network models have been developed based on the feed-forward/back-propagation algorithm and their performances compared to the KESALs predicted using the AASHO formula.

Neural network and multivariate statistics regression were used to an attempt to model the computed and predicted KESALs values. Of the 244 pavement sections, 234 sections were selected to achieve the aims of this study.

A complete set of average annual rain fall, average annual number of days below 32°F (0°C), drainage coefficients, observed PSI loss, back-calculated resilient moduli for the subgrade, structural numbers, and the subgrade types (Gravel, Sand, Silt, Clay, SiltyClay, Rock) were used for the prediction of the KESALs, using the AASHTO equation as well as those obtained from observed values.

An extremely accurate model using neural network modeling method was developed.

This NN model gave coefficient of correlations of 0.999 and 0.976 during the training stage, in models 1 and 2, respectively. Whereas it was not possible to model the data using multivariate linear statistic models with a coefficient of correlation of 0.68.

The neural network proved to be an extremely powerful tool for predicting KESALs (both predicted and observed). Similar NN models may be developed for other databases of LTPP's GPS program to include other types of pavement structures.

Throughout this paper, the database has been studied in order to re-evaluate the model for equivalent single-axle loads to cause the observed losses in PSI using the database presented in AASHO road test.

We found that the AASHO equation does not represent the cumulative equivalent single-axle loads. Significant discrepancies between the AASHO-predicted KESALs and the observed values have been modeled using the neural networks.

It has been shown that a local approximation feed-forward/back-propagation neural network can model the pavement performance data for the entire input domain better than a global approximation, such as the AASHTO formula.

The neural network method was used to build a nonparametric model for pavement performance using the road test data and its performance was compared with the AASHTO model.

In this study, NN design method for flexible pavements is proposed as a replacement for the AASHTO classical design equation.

This new model can represent the data much more accurately than the AASHTO formula. The AASHTO design overestimates ESALs whereas the proposed new model does not.

KESAL values can be estimated using artificial neural networks without the complex design equality of AASHTO. More importantly, the NN model gives the transfer coefficient from the AASHTO design estimation output to actual KESAL values occurred in test sections.

Thus, the design traffic values that can cause deterioration can be calculated more accurately using the NN model than with the AASHTO design equation.
